# Intranasal oxytocin in a genetic animal model of autism

**DOI:** 10.1038/s41380-023-02330-6

**Published:** 2023-12-15

**Authors:** Jakub Szabó, Matúš Mlynár, Andrej Feješ, Emese Renczés, Veronika Borbélyová, Daniela Ostatníková, Peter Celec

**Affiliations:** 1 Institute of Molecular Biomedicine, Faculty of Medicine, Comenius University, Bratislava, Slovakia; 2 Institute of Physiology, Faculty of Medicine, Comenius University, Bratislava, Slovakia; 3 Institute of Pathophysiology, Faculty of Medicine, Comenius University, Bratislava, Slovakia

**Keywords:** Autism spectrum disorders, Physiology

## Abstract

Autism spectrum disorder (ASD) is a group of neurodevelopmental disorders mainly characterized by deficient sociability and repetitive behaviors. Effective treatment for the core symptoms of ASD is still lacking. Behavioral interventions show limited effectiveness, while pharmacotherapy focuses on the amelioration of secondary symptomatology. Oxytocin (OXT) is a neuropeptide known for its prosocial impact, making it a candidate drug for ASD treatment. Its alleviating effect has been and still is widely researched, but outcomes reported by clinical studies are ambiguous. We examined the effect of daily intranasal OXT (0.8 IU/kg) administration for 4 weeks on the ASD-like phenotype in *Shank3*^*−/−*^ adult mice. Animals treated with OXT spent twice as much time interacting with the social partner as early as after 2 weeks of treatment. Furthermore, OXT-treated mice exhibited reduced explorative behavior by 50%, after 4 weeks of treatment, and a 30% reduction in repetitive behavior, 4 weeks after treatment termination. One-fold higher sociability and 30% reduced exploration due to OXT lasted up to 4 weeks following the treatment termination. However, social disinterest was elevated by roughly 10% as well, indicating a form of social ambivalence. Obtained results support the therapeutic potential of intranasally administered OXT in alleviating social shortfalls in a genetic model of ASD. Subsequent research is necessary to elucidate the benefits and risks of the long-term OXT administration, as well as its applicability in other ASD models and the potential treatment effect on social communication, which was not measured in the present study.

## Introduction

Autism spectrum disorder (ASD) is a group of neurodevelopmental disorders characterized by deficits in social interaction, communication, and repetitive behavior [1]. Since the etiopathogenesis is unknown, the treatment is symptomatic and inefficient. Only a limited number of ASD symptoms are treatable to this day. So far, the most prevalent treatment has been antipsychotic medication to manage irritability and hyperactivity [2]. Intense research is currently being done on novel treatment approaches targeting the core symptoms [3].

Oxytocin (OXT) is a neuropeptide best known for its crucial role in social bonding, social behavior, social recognition, and social care [4]. Previous research showed that OXT is implicated in neuroanatomical regions and neurochemical mechanisms responsible for processing social stimuli [5, 6] and its intranasal administration alters activation across brain regions of patients with ASD [7]. Furthermore, some studies have found that children suffering from ASD have lower concentrations of circulating OXT [8] and they were shown to be associated with annotated ASD risk genes [9]. Finally, a recent article showed that disruptions in OXT-related genes are associated with ASD etiology and may pose as potential biomarkers for predicting the efficacy of the OXT treatment [10]. These findings suggest its use as a possible therapeutic agent for several psychiatric disorders defined by deficits in sociability, such as ASD.

Several clinical studies have found that intranasal OXT administration improved social functioning and decreased repetitive behavior in patients suffering from ASD [11–14]. On the other hand, other studies failed to provide evidence supporting the therapeutic potential of intranasal OXT [15, 16], and a substantial number of clinical studies are still underway. However, OXT is still not being utilized due to its limited effectiveness, which appears to depend on the dosage, route of administration, and, most importantly, the characteristics of the individual patients undergoing treatment; this accents the need for further investigation of OXT as a possible therapeutic agent.

With the unclear etiopathogenesis and high heritability of ASD, genetic factors stand out as the main component involved. Thus, the genetic models of ASD pose an ideal choice for research into the disorder. One of the most prominent genetic animal models used in ASD research is the *SH3 and multiple ankyrin repeat domains 3* (*Shank3*) gene deficiency model, which exhibits a variety of ASD-like phenotypes, such as extensive grooming indicating repetitive behavior, or abnormal social behavior [17]. Mouse models carrying *Shank3* deficiency are widely preferred rodent models when it comes to ASD research. However, the research on the behavioral effects of OXT in the *Shank3* animal model so far has been almost exclusively limited to rats.

An attenuating role of an acute dose of OXT on ASD phenotype was shown in *Shank3*-deficient rats [18] and another paper reported normative concentrations of endogenous OXT in rats with a complete deletion of *Shank3* [19]. Morphological and structural neuronal deficits observed in mice carrying *Shank3* deficiency have been rescued with early-life acute subcutaneous OXT administration in a recent study [20]. Acute and chronic intranasal OXT administration was also reported to produce different behavioral effects [21]. Thus, considering a more universal therapeutic response is produced following chronic administration, it seems to be a preferred choice for the OXT treatment.

To elucidate the behavioral effects of chronic non-invasive OXT treatment on ASD symptomatology, we employed the *Shank3*-deficient mouse model of ASD. We expected daily treatment to alleviate social deficits and manage stereotypical behavior previously reported in *Shank3*-deficient mice. We anticipated such an effect to last even after the treatment termination, producing a long-term behavioral response.

## Material and methods

### Animals

Heterozygous breeding pairs of *Shank3B* mice with pure C57BL/6 background were obtained from The Jackson Laboratory (JAX Stock No. #017688). *Shank3B* wild-type (WT) and knock-out (KO) mutant mice were generated by crossing adult (3-month-old) heterozygous males with age-matched heterozygous females Genotyping was conducted to determine the genotype of offspring. WT (female=15, male=11) and *Shank3B*^*−/−*^ KO (female=12, male=16) adult (3-month-old) mice of both sexes with C57BL/6 background were used. Animals were group-housed (4-6 per cage) with their littermates and kept in a controlled environment of 24 ± 2 °C and 55 ± 10% humidity with *ad libitum* access to food and water on a 12-h light/dark cycle. The experiment was performed in accordance with the *Animal Research: Reporting of In Vivo Experiments (ARRIVE)* guideline [22]. Power analysis was conducted using G*Power. No animals were excluded from the study. Animals were randomized into treatment groups, but no blinding was conducted. The experiment has been conducted in accordance with the Slovak national laws and approved by the ethics committee of the Institute of Molecular Biomedicine.

### Genotyping

To confirm the genotype, end-point PCR was utilized. Tail samples (~0.2 cm) were collected from the animals at the time of weaning. Tail samples were then processed, and genomic DNA was isolated using the MyTaq^TM^ Extract-PCR Kit (Bioline) according to the kit’s instructions Following the extraction, 2 μl of the extract containing the DNA was amplified using standard Endpoint PCR and a combination of three primers designed to identify both the WT and KO alleles (P1-Common: GAG ACT GAT CAG CGC AGT TG; P2-WT: TGA CAT AAT CGC TGG CAA AG; P3-KO: GCT ATA CGA AGT TAT GTC GAC TAG G). Combinations of primers produced a 374-bp band for the WT allele, a 153-bp band for the KO allele, and both bands for the heterozygous allele. The PCR product was run on a 1.5% agarose gel stained with the GoodView^TM^ Nucleic Acid Stain (Beijing SBS Genetech Co., Ltd.).

### Substance administration

Animals were intranasally administered 0.8 IU/kg OXT (Ferring Pharmaceuticals, Switzerland) with the concentration of 1 IU/ml or saline vehicle treatment once daily between 12:00 and 15:00 according to the previously used protocol [23] for the period of 30 consecutive days. For the intranasal administration, a single channel 0.5 – 10 μl Pipette was used (Eppendorf, Germany). Drops of solution were gently placed equally on both nostrils of the mouse, which were inhaled. Administration and handling were consistent across groups and days of administration. No anesthesia was required, as the administration is non-invasive.

### Behavioral testing

Behavioral testing was conducted 12 h after the last daily substance administration throughout 2 consecutive days. Mice were tested in the PhenoTyper 4500 cage (Noldus Information Technology, Wageningen, The Netherlands) including an open field arena (45 cm × 45 cm × 45 cm) for 2 behavioral assays, the Reciprocal interaction test, and the Open field test. Each test was carried out in a dimly lit room, with a room temperature of 24 ± 1 °C. All animals were habituated to the room at least 30 min prior to each test. Mouse handling and experiments were carried out by the same experimenters throughout the study. After the behavioral testing, all animals were weighed.

In the *Reciprocal interaction test*, subject animals were socially isolated for 24 h prior to the testing and then randomly paired with a socially novel WT animal of the same sex used as a social partner. Both animals were placed in a cage filled with sawdust bedding and left to freely interact for 10 min while being recorded. The recording was manually scored by two observers blind to the experimental groups for cumulative time spent nose-to-nose, nose-anogenital, and side-sniffing as a measure of social interaction, and self-grooming, digging, lying flat, freezing in contact, or avoiding the social partner as a measure of social disinterest. The average values of both observations were calculated and considered representative. The inter-observer variability was 4.4%.

In the *Open field test*, animals were individually placed into the cage and left to freely explore for 10 min while recording. Recordings were manually scored by two observers blind to the experimental groups for a cumulative time of repetitive self-injurious grooming and rearing behavior as a measure of repetitive and explorative behavior, respectively. The average values of both observations were calculated and considered representative. The inter-observer variability was 3.9%. The distance the animals traveled during the test was quantified as a measure of locomotor activity and the time they spent in the central zone was quantified as a measure of anti-anxiety behavior.

### Statistical analysis

Statistical analyses were conducted using IBM SPSS Statistics 23.0 (IBM, Armonk, NY, USA). One-way ANOVA was used to compare the groups, followed by the post hoc analysis using Bonferroni correction. To elucidate the interactive effect of treatment and genotype, Two-way ANOVA was employed and to attest to the effect of time, Repeated Measures ANOVA was utilized with subsequent Bonferroni correction. Data are presented as mean ± SEM. *P*-values of less than 0.05 were considered significant. As no sex differences were observed, the animals were sex-pooled for statistical analysis.

## Results

### Sociability

**Fig. 1:**
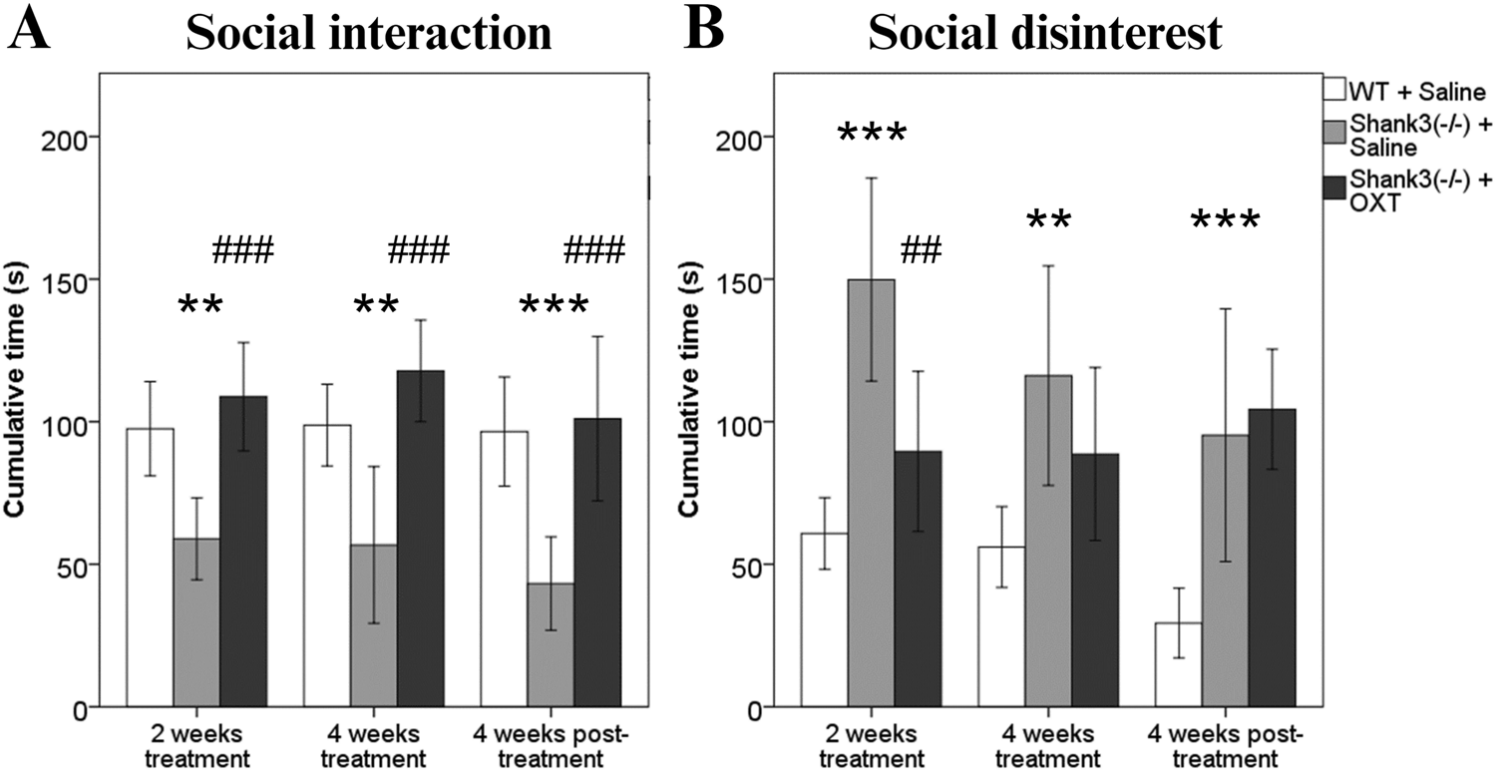
The effect of intranasal oxytocin on sociability. **A** Cumulative time spent socially interacting with a social-partner mouse in Reciprocal interaction test. **B** Cumulative time spent without interest in social contact with a social-partner mouse in Reciprocal interaction test. * 0.05; **/## *p* < 0.01; ***/### *p* < 0.001.

Social interaction differed between the groups of animals after 2 weeks of daily OXT administration [*F*(2, 53) = 6.55, *p* = 0.003]. Saline-treated *Shank3B*^*−/−*^ mice exhibited approximately half the time (58.9 s ± 6.4) interacting with the social-partner mouse compared to WT controls (97.5 s ± 8, *p* = 0.006, Fig. 1A) indicating the effect of genotype. A noteworthy effect of treatment was observed as well. *Shank3B*^*−/−*^ mice that received OXT, compared to their saline-treated *Shank3B*^*−/−*^ controls, exhibited double the time socially interacting with a partner (108.7 s ± 8.9, *p* < 0.001, Fig. 1A). Similar results were observed in social interaction after 4 weeks of OXT treatment between the groups [*F*(2, 53) = 9.55, *p* < 0.001]. *Shank3B*^*−/−*^ mice treated with saline consistently showed deficits in sociability (56.7 s ± 12.3) with roughly 50% less time spent interacting with a social-partner animal than the WT control mice did (98.7 s ± 7, *p* = 0.003, Fig. 1A). Furthermore, *Shank3B*^*−/−*^ animals treated with OXT interacted with the social partner twice as much compared to the saline-treated *Shank3B*^*−/−*^ mice (117.8 s ± 8.4, *p* < 0.001, Fig. 1A). Finally, when the animals were behaviorally tested 4 weeks following the OXT treatment termination, distinct differences between the groups were observed in social interaction also [*F*(2, 53) = 6.08, *p* = 0.004]. Saline-treated *Shank3B*^*−/−*^ controls displayed half the time interacting with a socially novel mouse (43.2 s ± 7.3) compared to what was detected in WT controls (96.5 s ± 9.3, *p* = 0.001, Fig. 1A). Most importantly, OXT-treated *Shank3B*^*−/−*^ mice exhibited over twice as much time spent in social interaction than saline-treated *Shank3B*^*−/−*^ mice (101.1 s ± 13.6, *p* = 0.001, Fig. 1A). No effect of time was observed in social interaction.

The results obtained when quantifying social disinterest indicate mostly complimentary outcomes to the ones observed in social interaction. During the measurement after 2 weeks of daily administration, animal groups differed significantly in disregard towards the social partner mouse in the Reciprocal interaction test [*F*(2, 53) = 15.66, *p* < 0.001]. *Shank3B*^*−/−*^ mice treated with saline spend almost three times more time avoiding contact with a partner mouse (149.8 s ± 16) than the WT mice (60.8 s ± 6.1, *p* < 0.001, Fig. 1B). On the other hand, OXT-treated *Shank3B*^*−/−*^ mice exhibited roughly 50% less time spent avoiding the social partner compared to controls (89.6 s ± 13.2, *p* = 0.008, Fig. 1B). When looking at the results from the measurement after 4 weeks of daily OXT administration, once again a significant difference between groups was recorded [*F*(2, 53) = 6.52, *p* = 0.003]. Saline-treated *Shank3B*^*−/−*^ mice spent 25% more time not interested in their social-partner mouse (116.1 s ± 17.3) than the WTs (56 s ± 6.9, *p* = 0.006), while *Shank3B*^*−/−*^ mice treated with OXT did not significantly differ from their *Shank3B*^*−/−*^ controls (88.6 s ± 14.3, *p* = 0.234, Fig. 1B). Upon investigating the social disinterest 4 weeks following the treatment termination, groups differed considerably [*F*(2, 53) = 18.96, *p* < 0.001]. A large part of this difference was made up of saline-treated *Shank3B*^*−/−*^ mice, which spend more than three times longer disregarding the social-partner mouse (95.2 s ± 19.9) compared to the WT mice (29.4 s ± 5.9, *p* < 0.001). No significant differences were shown between *Shank3B*^*−/−*^ mice treated with OXT and *Shank3B*^*−/−*^ mice treated with saline. Finally, the interactive effect of grouping and time was observed [*F*(2, 51) = 8.89, *p* < 0.001] across the animal groups. Both WT mice and *Shank3B*^*−/−*^ mice treated with saline exhibited a consistent decrease in social disinterest in time, however, this was not true for the OXT-treated *Shank3B*^*−/−*^ mice which exhibited an increasing tendency over the testing time points [*F*(2, 32) = 6.76, *p* = 0.019].

### Repetitive behavior

**Fig. 2:**
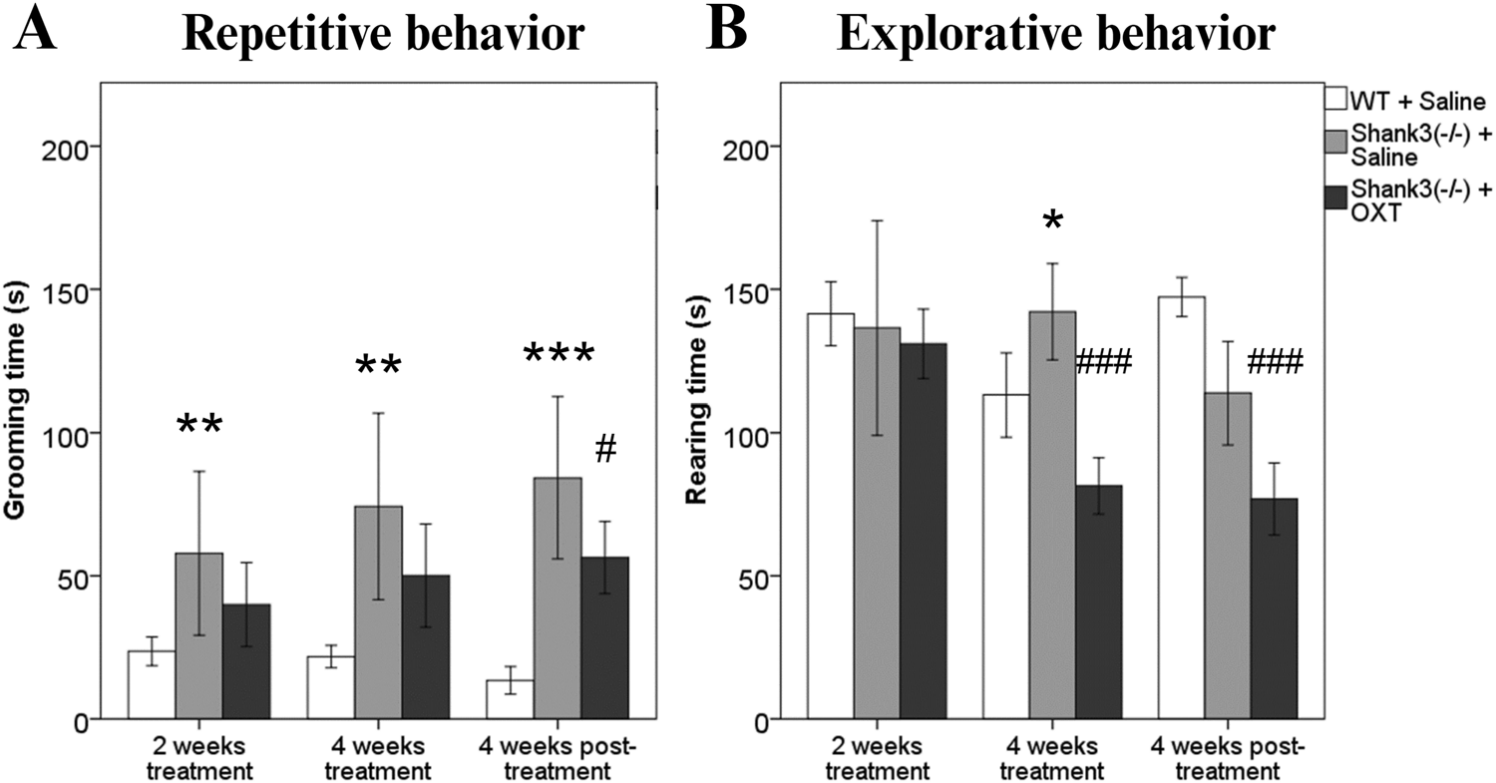
The effect of intranasal oxytocin on repetitive and explorative behavior. **A**. Cumulative time spent self-grooming in the Open field test. **B**. Cumulative time spent rearing in Open field test. */# *p* < 0.05; **/## *p* < 0.01; ***/### *p* < 0.001.

The repetitive behavior differed between the groups after 2 weeks of treatment [*F*(2, 51) = 6.90, *p* = 0.002]. A main effect of genotype was observed, as the saline-treated *Shank3B*^*−/−*^ mice exhibited over twice the amount of self-injurious grooming (57.8 s ± 12.9) compared to the WT controls treated with saline (23.6 s ± 2.4, *p* = 0.002, Fig. 2A). However, *Shank3B*^*−/−*^ mice treated with OXT did not differ when compared to their *Shank3B*^*−/−*^ controls treated with saline (39.9 s ± 6.9, *p* = 0.194). Data analysis at 4 weeks since the treatment started revealed differences between groups in repetitive behavior as well [*F*(2, 51) = 12.99, *p* < 0.001]. *Shank3B*^*−/−*^ mice treated with saline spend 70% more time grooming (74.3 s ± 14.6) than the WT controls (21.8 s ± 1.9, *p* = 0.005, Fig. 2A). Once again, OXT-treated *Shank3B*^*−/−*^ mice did not exhibit reduced self-grooming compared to the saline-treated *Shank3B*^*−/−*^ control mice (50.1 s ± 8.5, *p* = 0.137). Finally, 4 weeks following the treatment termination, the difference between the groups was recorded [*F*(2, 51) = 36.56, *p* < 0.001]. Similar to the previous time points, saline-treated *Shank3B*^*−/−*^ mice exhibited 5-times more self-grooming (84.2 s ± 12.7) than the WT controls (13.4 s ± 2.3, *p* < 0.001, Fig. 2A). Interestingly, this time the OXT-treated *Shank3B*^*−/−*^ mice exhibited reduced self-grooming by a third compared to the saline-treated *Shank3B*^*−/−*^ mice (56.3 s ± 5.9, *p* = 0.036, Fig. 2A). However, no effect of time was implicated in this finding [*F*(2, 32) = 2.38, *p* = 0.108].

### Explorative behavior

Two weeks after OXT administration, groups did not differ in explorative behavior [*F*(2, 32) = 0.49, *p* = 0.616]. However, after 4 weeks of treatment, a difference was observed between the groups [*F*(2, 32) = 14.3, *p* < 0.001]. Saline-treated *Shank3B*^*−/−*^ mice spend about 20% more time exploring their surroundings than the WT controls (113.1 s ± 7.1, *p* = 0.021, Fig. 2B). Furthermore, OXT-treated *Shank3B*^*−/−*^ mice exhibited almost half as much time exploring compared to the saline-treated *Shank3B*^*−/−*^ mice (81.4 s ± 4.62, *p* < 0.001). Similarly, explorative behavior differed between the groups even 4 weeks after the treatment termination [*F*(2, 32) = 54.4, *p* < 0.001]. This time, saline-treated *Shank3B*^*−/−*^ mice exhibited about 20% lower explorative behavior (113.7 s ± 8.1) compared to the WT controls (147.3 s ± 3.3, *p* = 0.002, Fig. 2B). Most importantly, *Shank3B*^*−/−*^ mice treated with OXT exhibited 30% less interest in exploring their surroundings when compared to the saline-treated *Shank3B*^*−/−*^ control mice (76.9 s ± 5.9, *p* < 0.001, Fig. 2B).

### Anxiety-like behavior

**Fig. 3:**
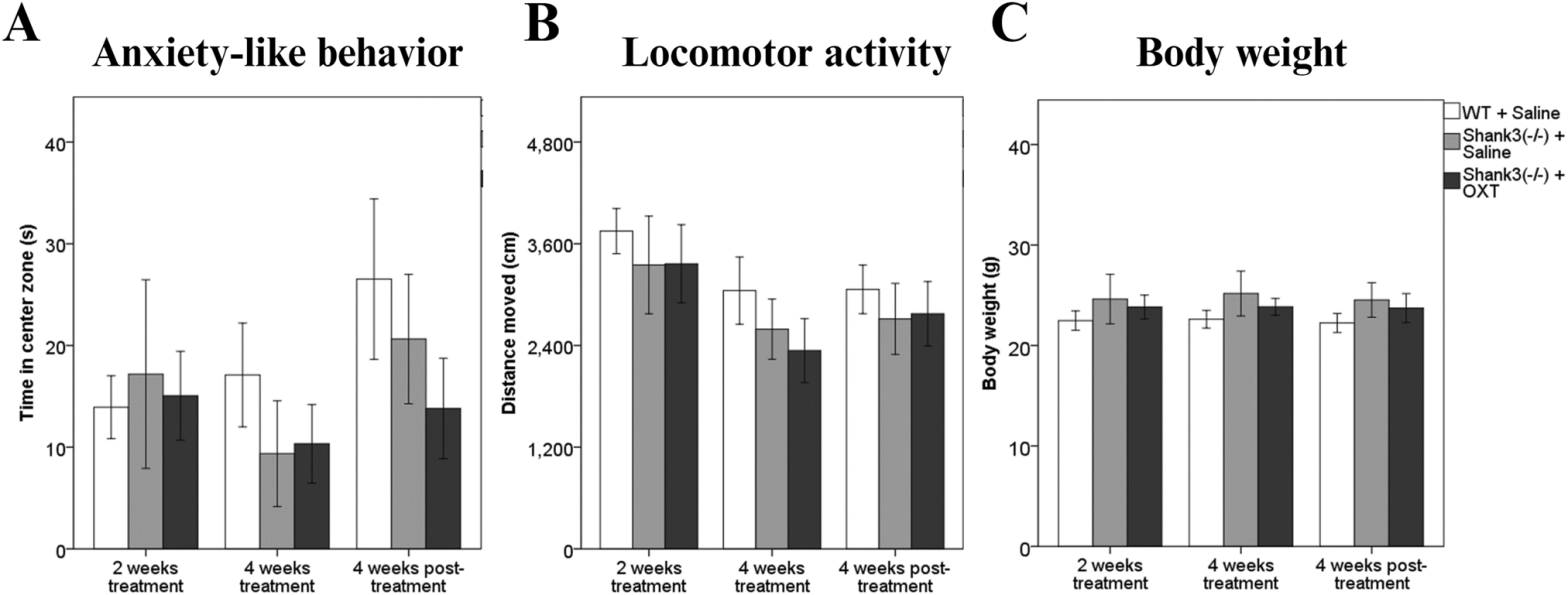
The effect of oxytocin treatment on anxiety-like behavior, locomotor activity and body weight. **A** Cumulative time spent in the center zone in the Open field test. **B** Cumulative distance traveled in Open field test. **C**. Body weight.

No differences between the groups were observed in anxiety-like behavior either after 2 weeks of treatment [F(2, 51) = 0.46, *p* = 0.632], or after 4 weeks of treatment [F(2, 51) = 3.21, *p* = 0.481], or 4 weeks following the treatment termination [F(2, 51) = 3.57, *p* = 0.352, Fig. 3A].

### Locomotor activity and body weight

The animals did not differ in locomotor activity after 2 weeks [*F*(2, 51) = 1.68, *p* = 0.195], and 4 weeks of treatment [*F*(2, 51) = 3.89, *p* = 0.271], or 4 weeks after the treatment was suspended [*F*(2, 51) = 1.34, *p* = 0.269, Fig. 3B]. Finally, the groups did not differ in body weight at any time points: after 2 weeks [*F*(2, 51) = 2.94, *p* = 0.062], or 4 weeks of the treatment [*F*(2, 51) = 3.19, *p* = 0.49], and 4 weeks after the administration was terminated [*F*(2, 51) = 2.92, *p* = 0.063, Fig. 3C].

## Discussion

This study was, to our knowledge, the first to examine the effect of chronic intranasal OXT treatment on autistic phenotype in the *Shank3B*^*−/−*^ mutant mouse model of ASD. Daily intranasal administration of OXT prevented social deficits exhibited by *Shank3B*^*−/−*^ mice as early as after 2 weeks of treatment, but further manifested in a form of social ambivalence. Treated animals exhibited a reduced interest in their surroundings and slightly attenuated repetitive self-injurious behavior 4 weeks after the treatment was terminated.

Amelioration of social behavior due to OXT in the current study is a finding consistent across several previous studies with BALB/cByJ and C58/J mice models of ASD [24, 25] despite their variations in volumes, lengths, and routes of administration, which might produce different prosocial effects [21]. However, in the BTBR mouse model [23], with which our study shared a substantial portion of methodology, such as administration protocol, dose, and behavioral assays, no effect of OXT treatment on sociability was recorded. The differences in results could be partly explained by the disparity of age in which the animals were tested in both studies; our study was done on adult mice, whereas Bales et al. [23] used mice in adolescence. Furthermore, the authors claim to observe no effects of long-term exposure to OXT on social interaction but assessed the reciprocal social interaction only 3 days following the daily OXT administration, starting at weaning. It is thus plausible that the substance in the presented dose did not take a sufficient effect at the time of assessment. A recent study investigating the effects of OXT administered by intracranial injections in *Shank3*^*−/−*^ rats has noted an impairing effect on social memory, but not on social interaction [18]. Presumably, the differences in the administration route as well as in the genetic background of subjects might be causing disparities in the results. Finally, a recent study recorded no effect of intranasal OXT treatment on treating the sociability deficit across three different mouse models of autism, including the *Shank3B*^*−/−*^ mutant mice [26]. However, it is important to pinpoint that the Three-chamber social interaction test was utilized to quantify sociability in the article. Several previous studies reported the Three-chamber social interaction test not being able to intercept social deficits, whereas the Reciprocal interaction test was [27, 28]. Thus, the validity of the assay could be in question and can play a role in the disparity of recorded results in sociability.

Furthermore, we observed a steady increase in social disinterest in OXT-treated animals across the testing time points. This finding suggests a form of socially ambivalent behavior, where on one hand, daily administration of OXT ameliorated social deficits by making the animals more interested in a social partner, but on the other hand, it also steadily increased social irritability following contact with the social partner as well. Such duality was not yet reported in similar research, so we can only hypothesize about the mechanisms at play. There is evidence pointing to changes in OXT receptor expression and regulation following the stimulation by the exogenous neuropeptide [23]. In line with this, it has been reported earlier that the stimulation of OXT receptors increases the expression of neurexins and scaffolding proteins, such as SHANK3 in a cell-specific manner [29], rescuing the deficits caused by the mutation to some degree. However, pharmacologically increased OXT availability over a longer period is known to lead to receptor downregulation, which can be manifested in paradoxical effects [30–32]. Thus, presumably, it can be the cause of the observed socially ambivalent behavior. However, as we did not record the expression of the OXT receptors in the brain, further research is required to elucidate this hypothesis.

Repetitive behavior was consistently impaired in *Shank3B*^*−/−*^ mice in our study and the OXT administration only partially ameliorated the deficit. The attenuation of extensive repetitions in animals treated with OXT was observed only 4 weeks after the daily treatment was terminated. Interestingly though, such an outcome could shed some new light on inconclusive findings provided in literature so far. It was previously reported that acute OXT treatment in young adult C58/J mice decreased repetitive grooming [25], and similarly to this, in a valproic acid-induced rat model of autism, repetitive behavior was rescued by OXT administration [33]. Besides the differences in the age of subjects, mentioned studies used intraperitoneal and subcutaneous routes of administration, respectively, which are reported to provide a more systemic substance effect [34]. On the other hand, using the intranasal route of OXT administration for 7 successive days does not seem to mediate such a therapeutic effect on repetitive behavior [23, 35]. Therefore, this evidence points to a possibility that for OXT to influence repetitive behavior, a more systemic effect needs to be induced, possibly targeting relevant underlying mechanisms not yet elucidated. In addition, it could be presumed that intranasal OXT is fully able to impact excessive repetitive behavior if delivered consistently over a longer period. Taken together, the route and time of administration can arbitrate an attenuating effect of OXT on repetitive behavior.

Animals that received OXT daily in our experiment exhibited a reduced interest in exploring their surroundings following administration over a longer period. Previously reported effects of chronic OXT increasing the exploration [21] were not supported by our results. As the effect in the mentioned paper was dose-dependent, i.e., only observed in the group with an OXT dose of 5 IU/kg, which is an over 5-fold higher dose than the one used in our study, we expect the lower dose to be the key factor behind the noted discrepancies. There is some evidence reporting OXT having sedative effects in rhesus monkeys and rabbits [36], but only a trend of mild sedation was observed in research with BTBR mice receiving OXT treatment [23]. Although the OXT-treated *Shank3B*^*−/−*^ mice in our experiment exhibited reduced explorative behavior mediated by treatment, they showed normative locomotion, anxiety-like behavior, and body weight change. This suggests the possible OXT-induced sedative effect we observed was not neophobia- or activity-driven, but rather specifically reduced interest in the surroundings of the animal. Subsequent research into this is therefore needed.

Our findings, however, should be understood with several limitations in mind. The concentrations of the absorbed OXT, as well as its kinetics, were not recorded, therefore we lack information on effect duration. Furthermore, as ASD is primarily prevalent at an early age, adulthood might not be the most optimal age for modeling the disorder. Although we set out to investigate the OXT effect on the core ASD symptomatology, we lacked any measure for the deficits in communication, which are highly prevalent in autism. On the other hand, as impaired sociability is arguably the most debilitating condition of ASD, we illustrated a substantial attenuative effect of the substance, rendering the social deficiency to none.

To what degree the reported results can be translated to human research and if at all, relies on clinical studies. Recently published results from clinical trials suggest little to no therapeutic effect of OXT in patients with autism [16, 37–39], but others show dose-dependent [14], and age-dependent effects [40]. Given the heterogeneity as vast as in ASD, it is highly probable the same therapeutic reagent would not apply to all patients. We demonstrated that chronic intranasal OXT exposure can reliably ameliorate social deficits in the *Shank3B*^*−/−*^ mouse model of autism as early as after 2 weeks of administration. Furthermore, chronic treatment seems to have the potential to reduce extensive repetitive behavior as well. While the SHANK3 mutation is prevalent in only about 1% of patients with ASD [41], intranasal OXT treatment might pose an effective treatment option specifically for this subset.

## Data Availability

Data from this study are available from the authors upon reasonable request.
